# Role of *cis-trans* proline isomerization in the function of pathogenic enterobacterial Periplasmic Binding Proteins

**DOI:** 10.1371/journal.pone.0188935

**Published:** 2017-11-30

**Authors:** Paulina Cortes-Hernandez, Lenin Domínguez-Ramírez

**Affiliations:** 1 Centro de Investigacion Biomedica de Oriente (CIBIOR), Instituto Mexicano del Seguro Social (IMSS), Metepec, Puebla, Mexico; 2 Chemical and Biological Sciences, Universidad de las Américas Puebla (UDLAP), Cholula, Puebla, Mexico; Danish Cancer Society Research Center, DENMARK

## Abstract

Periplasmic Binding Proteins (PBPs) trap nutrients for their internalization into bacteria by ABC transporters. Ligand binding triggers PBP closure by bringing its two domains together like a Venus flytrap. The atomic determinants that control PBP opening and closure for nutrient capture and release are not known, although it is proposed that opening and ligand release occur while in contact with the ABC transporter for concurrent substrate translocation. In this paper we evaluated the effect of the isomerization of a conserved proline, located near the binding site, on the propensity of PBPs to open and close. ArgT/LAO from *Salmonella typhimurium* and HisJ from *Escherichia coli* were studied through molecular mechanics at two different temperatures: 300 and 323 K. Eight microseconds were simulated per protein to analyze protein opening and closure in the absence of the ABC transporter. We show that when the studied proline is in *trans*, closed empty LAO and HisJ can open. In contrast, with the proline in *cis*, opening transitions were much less frequent and characterized by smaller changes. The proline in *trans* also renders the open trap prone to close over a ligand. Our data suggest that the isomerization of this conserved proline modulates the PBP mechanism: the proline in *trans* allows the exploration of conformational space to produce trap opening and closure, while in *cis* it restricts PBP movement and could limit ligand release until in productive contact with the ABC transporter. This is the first time that a proline isomerization has been related to the control of a large conformational change like the PBP flytrap mechanism.

## Introduction

Periplasmic-binding proteins (PBPs) are a large family of structurally related proteins that bind diverse ligands from ions to saccharides to aminoacids for their import into gram-negative bacteria [1]. PBPs are composed of two domains that open and close around a hinge and this motion is a paradigm of large reversible conformational changes in proteins. This motion has been compared to the closing of the two lobules of a Venus flytrap over a prey [1]. Despite a lot of structural evidence to support the mechanism, the atomistic details that control PBP opening and closure for nutrient capture and release are not completely understood.

PBPs diffuse in the bacterial periplasm where they trap their specific ligand, functioning as the soluble counterparts of ABC importers. Ligand binding between domains triggers PBP closure through induced fit and conformational selection [2]. Then, nutrient-loaded PBPs bind their cognate ABC transporter, which hydrolyzes ATP and releases the ligand into the cytoplasm [3]. Crystallographic evidence for the maltose binding protein suggests that interaction with the ABC transporter is necessary for ligand release [4]. This structural information is lacking for other PBPs. Based on biochemical data [5] and on the limited structural evidence [4], it has been proposed that the interaction with the ABC transporter operates PBP opening concurrent with ATP hydrolysis. However, details for the PBP opening mechanism and for its coupling to the ABC transporter remain unknown. PBP opening and closure events have been difficult to pinpoint in molecular dynamics simulations [2,6–8]. Especially PBP opening has been elusive to observe in simulations lasting up to 200 ns with different force fields [2,9,10].

We report a computational study on two ortholog PBPs, LAO (also known as ArgT) from *Salmonella typhimurium* and HisJ from *Escherichia coli* that share 70% sequence identity (excluding signal peptides). Both PBPs bind histidine, lysine, arginine and ornithine and use the same ABC transporter, the HisQMP_2_ permease [11,12]. Structures for both PBPs have been determined by X-ray diffraction (XRD) and nuclear magnetic resonance (NMR) in two main forms: with ligands, in the so-called closed "holo-form" [11,13] and empty, in the open "apo-form" [14,15]. No intermediates have been solved. The RMSD between the closed and open states is 5.3 Å for LAO and 9.8 Å for HisJ over 238 Cα atoms.

We tested the effect of the isomerization state of a conserved proline on the propensity of these PBPs to open or close in molecular simulations, in the absence of their cognate ABC transporter. We found that the proline in *trans* allows for opening and closure of the trap, whereas in *cis* it limits PBP movement, suggesting that this proline modulates the trap mechanism.

## Results

**Fig 1:**
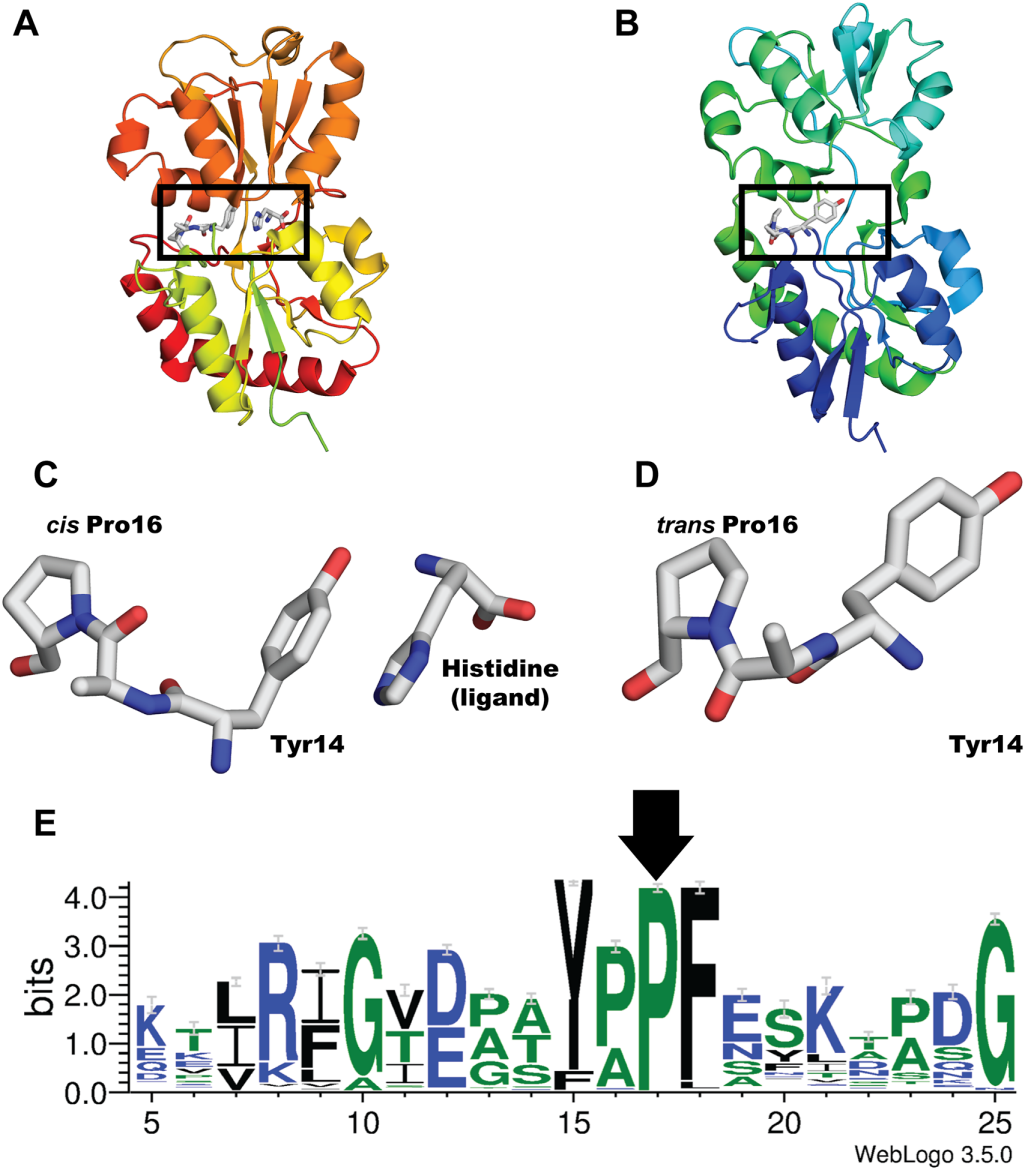
HisJ structural analysis reveals that conserved proline 16 displays different isomeric states in closed *versus* open structures. In (A) and (B) are ribbon representations of the solved structures for closed/with ligand (1HSL) and open/empty (2M8C) HisJ. Residues Tyr14, Ala15 and Pro16 are in sticks. (C) and (D) are close-ups to the boxed regions in A and B, pointing out the differences in residue conformation: the closed/with ligand structure has a *cis* Pro16 (A, C) while the open/empty form has a *trans* Pro16 (B, D). The isomerization state changes the position of Tyr14's side chain. (E) Shows the residue conservation in 450 PBP sequences obtained and aligned as described in Materials. Only non-redundant sequences with more than 30% and less than 95% homology were used. Pro16 (arrow) and 20 neighboring residues are shown.

Comparison of the HisJ closed/with-ligand structure (1HSL) against the open/empty (2M8C) revealed that the closed form has proline 16 (Pro16) in *cis* (Fig 1A and 1C) whereas the open form has it in *trans* (Fig 1B and 1D). This proline is conserved across at least 450 non-redundant PBPs (Fig 1E, arrow) and it has been solved in *cis* in all the LAO and HisJ structures reported so far, except for the aforementioned open 2M8C. The conformation of Pro16 in HisJ perturbs the orientation of binding site residue Tyr14 in the 3D structures (Fig 1C vs 1D), potentially affecting the interaction with the ligand. Hence, we tested the effect of the isomerization state of this conserved proline on the frequency of opening and closure events for LAO and HisJ. We performed sets of 50 ns simulations, at 300 K (26.85°C) and 323 K (49.85°C), starting from open/empty, open/with ligand, closed/with ligand and closed/empty structures for LAO and HisJ, with Pro16 computationally set to *cis* or *trans*. We ran the simulations 10 times per state, thus each PBP was sampled for 4 μs at each temperature (total simulated time was 16 μs in 400 simulations). A compilation of the simulations performed per condition is shown in S1 Table. No spontaneous proline isomerizations were observed during simulation.

### Opening transitions for LAO and HisJ can be detected at 300 K

**Fig 2:**
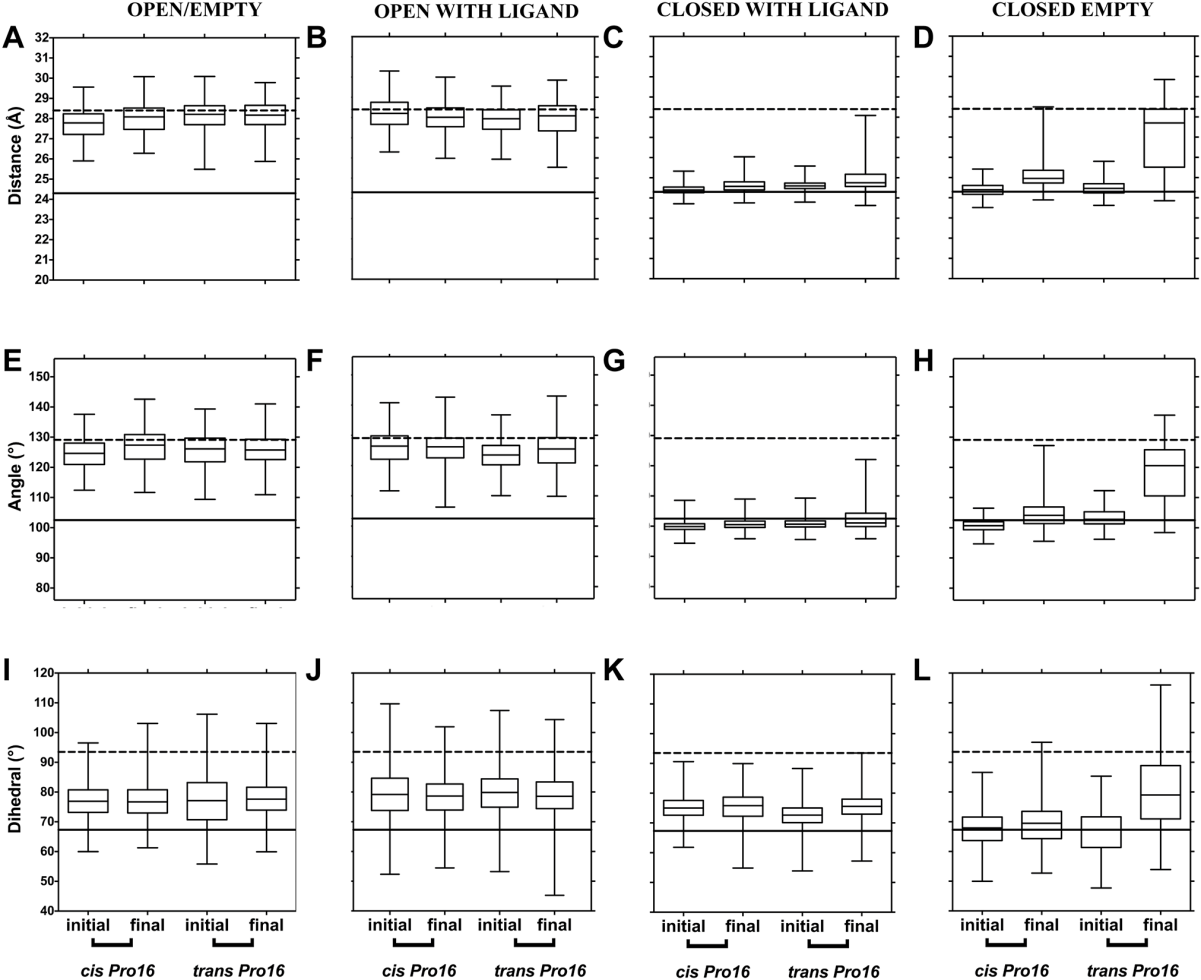
LAO domain separation during simulation at 300 K, monitored through distances, angles and dihedral between domains as a function of Pro16 isomerization state. (A-D) show distance, (E-H) show angles (I-L) show dihedral angles between domains. The starting structure used for each set of simulations is indicated at the top of each column. Boxes represent quartiles (upper, median and lower), while whiskers are maximum and minimum values for the initial and the final 5 ns of each 50 ns simulated. One value point was extracted for every 20 picoseconds of simulation, thus 2,500 values are represented in each box. Dotted and solid lines across the graphs represent the reference values for the open and closed states, respectively, calculated from structures PDB ID 2LAO (open) and PDB ID 1LAF (closed).

**Fig 3:**
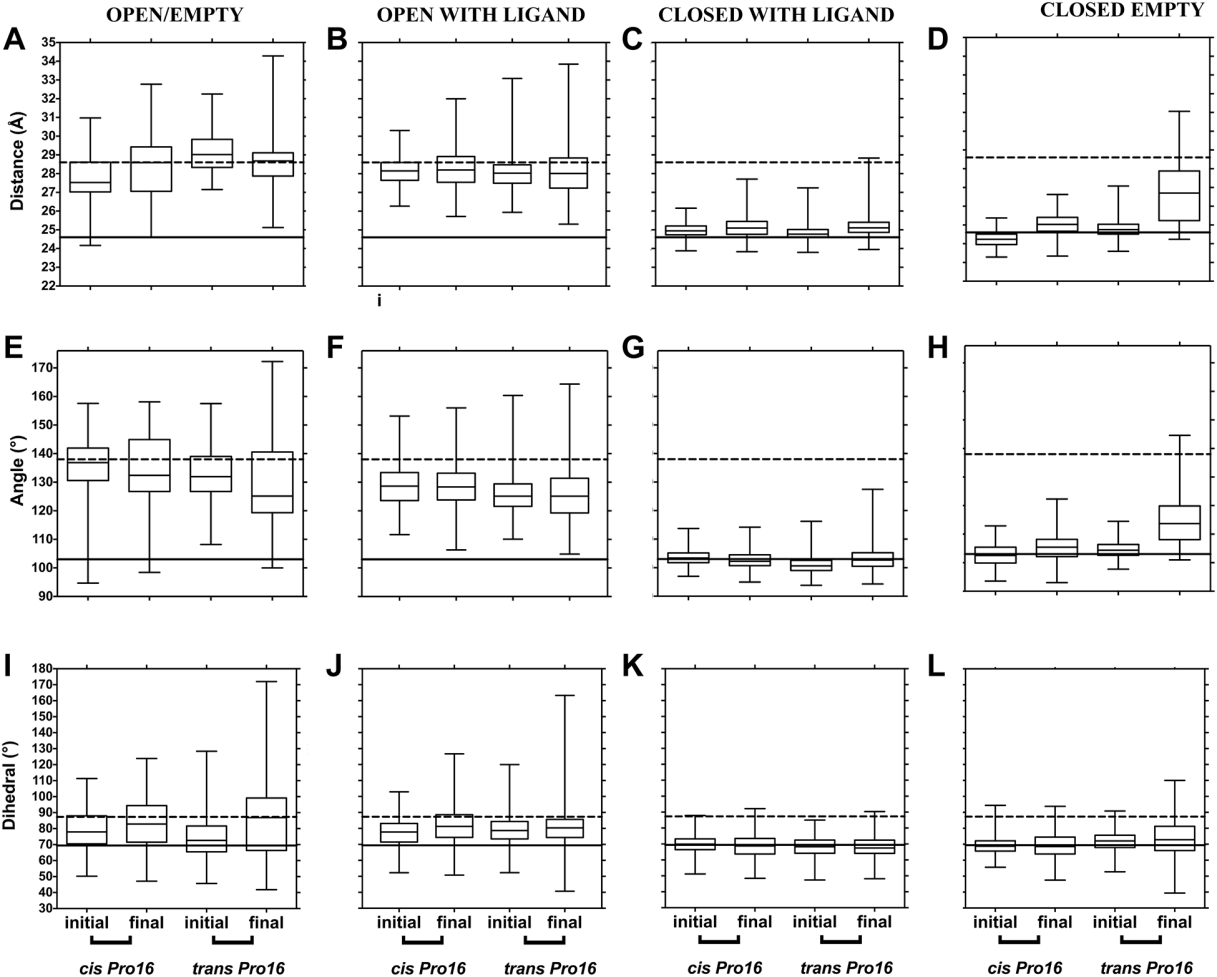
HisJ domain separation during simulations at 300 K, monitored through distances, angles and dihedrals between domains as a function of Pro16 isomerization state. Data were obtained, processed and presented as in Fig 2. Dotted and solid lines across the graphs correspond to the reference values calculated from open (PDB ID 2M8C) or closed (PDB ID 1HSL) structures.

First, we describe results with three conventional metrics of domain separation that have been used for PBPs: distance, angle and dihedral angle between domains' centers of mass (diagrams for these metrics are shown in S1 Fig and in [10]). To identify whether the PBPs changed during simulation, the values for the initial and the final 5 ns at 300 K were plotted (Fig 2 for LAO and Fig 3 for HisJ). Simulations that started from open PBPs (open/empty and open/with ligand) clustered with their respective open reference (dotted line) and displayed little change from the initial state (see first two columns in Fig 2 for LAO and Fig 3 for HisJ). This suggests that at 300 K, the open PBPs stayed open during simulation with no difference induced by Pro16 isomers. The open/with ligand structures retained the ligand for roughly 60% of simulation time, before releasing it into the solvent.

In turn, simulations starting from closed/with ligand conformations (third column in Figs 2 and 3) remained near the closed XRD reference (solid line) suggesting that, in presence of their ligand and regardless of the Pro16 isomer, these PBPs did not open during the 50 ns of simulation at 300 K. The top whisker of the final structures was longer and reached towards the open reference only with *trans* Pro16, implying more dispersion in that condition (closed/with ligand, C, G and K in Figs 2 and 3); but quartiles remained near the closed reference. In contrast, a change that depended on the Pro16 isomer was observed in the simulations that started from closed/empty PBPs. The values for the initial 5 ns clustered near the solid line of the closed reference but by the end of the simulation, those with *trans* Pro16 approached the dotted line of the open conformation, more so than those with *cis* Pro16 (last column in Figs 2 and 3). This suggests that at 300 K, in the absence of ligand, the sole isomerization of Pro16 from *cis* to *trans* allows larger separation between domains, enabling the closed structures to reach values similar to those of the open reference conformations.

To illustrate the extent of this putative opening, we plotted the RMSD of representative LAO simulations that started from the closed/empty state with Pro16 in *cis* or *trans* (S2A Fig). Although both structures displayed similar initial RMSD values, for *cis* Pro16 the RMSD remained stable throughout simulation, whereas for *trans* it increased after a lag and doubled the value observed for *cis*. The increase in RMSD (S2A Fig) and the metrics in Fig 2 are compatible with LAO opening with *trans* Pro 16 but not with *cis*. The extent of the separation between domains is observable in the structure shown in S2B Fig.

### Opening and closing transitions for LAO and HisJ are detectable at 323 K

Only the opening but not the closure transition was detected in PBP simulations at 300 K. To search for closing events, we conducted the simulations at higher temperatures to increase sampling. At the physiological temperature of 310 K (36.85°C) results were similar to 300 K (26.85°C) with changes consistent with opening but not with closure. Thus, we raised the temperature to 323 K (49.9°C). First, we examined if denaturation occurred at any of the simulated temperatures using the native contact Q [16–18]. For clarity, in this manuscript we will refer to this metric as Q(NC), for "Native Contacts". We plotted Q(NC)_open_ vs Q(NC)_closed_ (subscript describes the native reference structure used for calculation) (S3 Fig for LAO and S4 Fig for HisJ). Q(NC) values above 0.75 [19] suggest that the native states of LAO and HisJ were not disrupted during simulations at 300 or 323 K.

**Fig 4:**
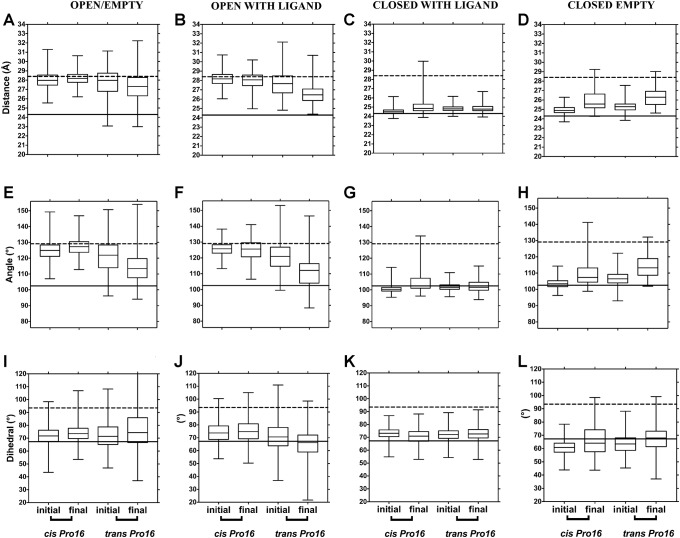
LAO domain separation during simulations at 323 K, monitored through distances, angles and dihedral between domains, as a function of Pro16 isomerization state. Data were obtained, processed and presented as in Fig 2.

**Fig 5:**
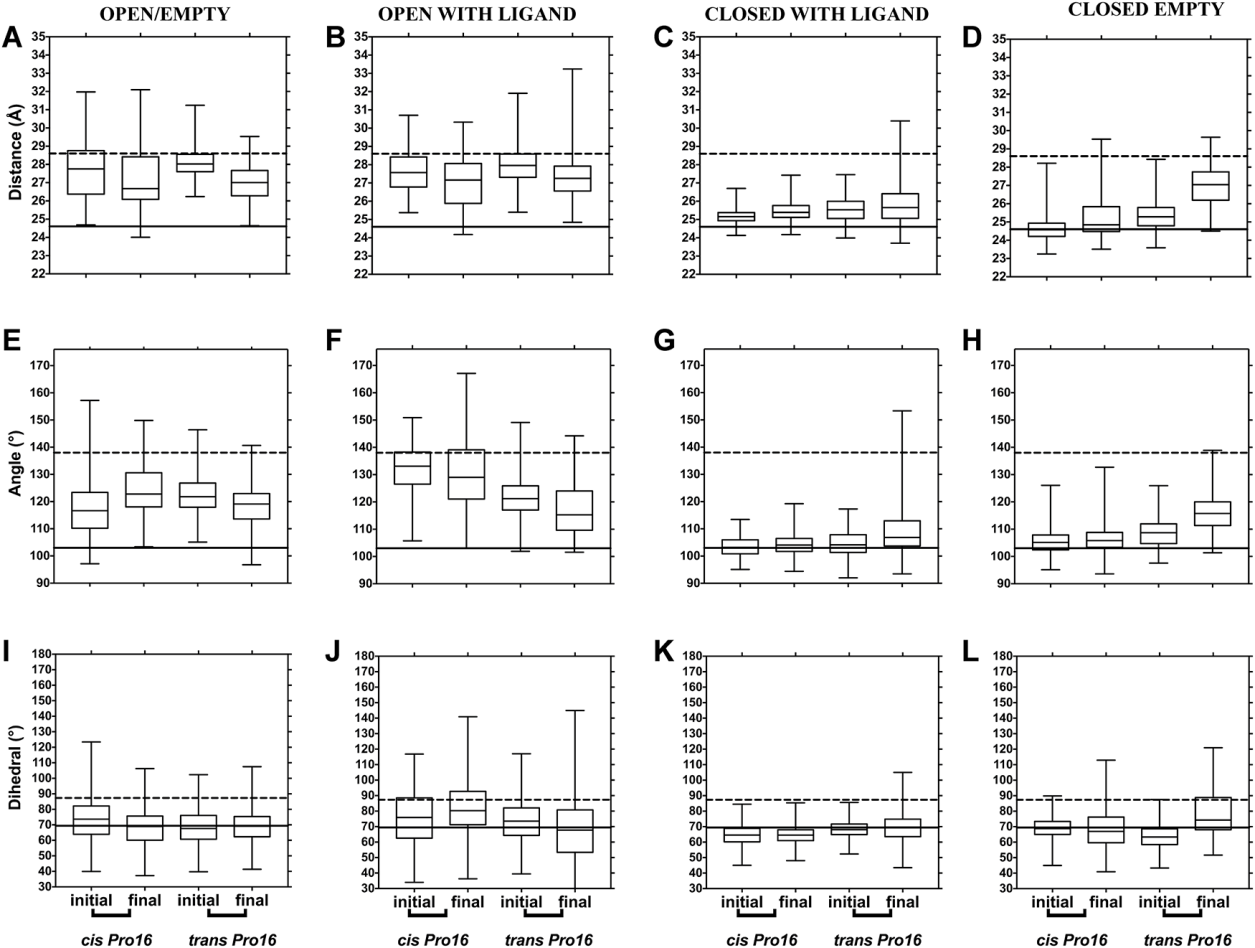
HisJ domain separation during simulations at 323 K, monitored through distances, angles and dihedral between domains as a function of Pro16 isomerization state. Data were obtained, processed and presented as in Fig 3.

Next, domain separation at 323 K was analyzed (Fig 4 for LAO and Fig 5 for HisJ) as in Figs 2 and 3. Closed states behaved similarly at 323 and 300 K: changes suggestive of opening of closed/empty structures were observed with *trans* Pro16. However, open states displayed changes at 323 K, suggestive of domain closure that hadn't been observed at 300 K: the final values for distance and angle between domains decreased, becoming similar to those of the closed crystallographic reference only with *trans Pro16* (see first two columns in Figs 4 and 5). This was more prominent for open/with ligand than for open/empty trajectories. Similar to what was observed at 300 K, at 323 K no large changes between initial and final values were detected with *cis* Pro16.

At 323 K, the RMSD of representative LAO simulations starting from the open/with ligand state, increased more with Pro16 in *trans* than in *cis* (S2C Fig) and domains came closer together with *trans* Pro16 in the final structures (S2D Fig), consistent with PBP closure. Overall, our analyses suggest that the *trans* isomer of Pro16 allows larger domain mobility, facilitating both closing and opening of the PBPs.

### Analysis of concatenated trajectories with multiple metrics refines the detection of PBP opening and closure events

**Fig 6:**
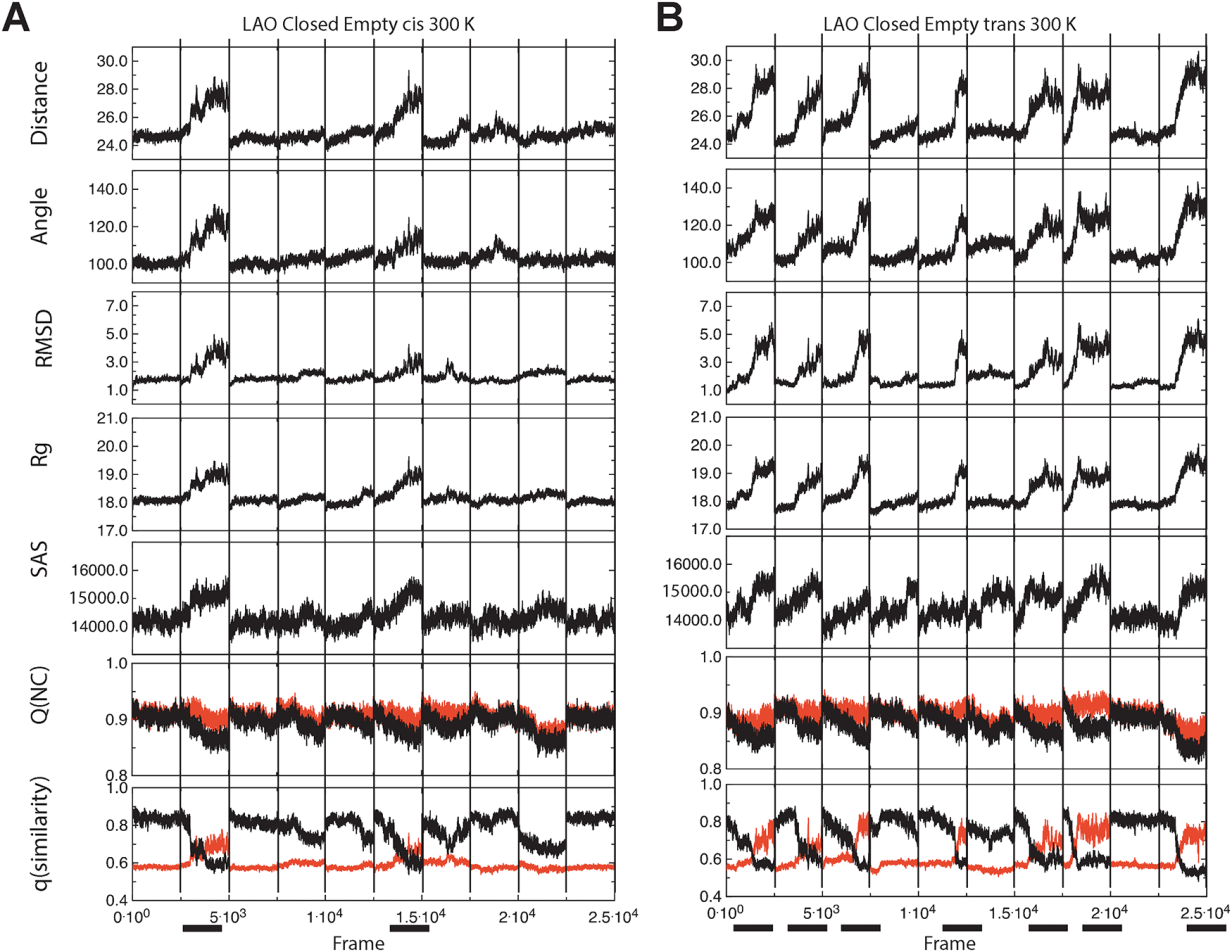
Changes in seven metrics during simulations of the closed/empty LAO with *cis* or *trans* Pro16 at 300 K. Ten different trajectories were concatenated and changes in distance, angle, RMSD, Rg, SAS, Q(NC) and q(similarity) were calculated. Each trajectory is separated by a vertical line. Distances and angles were measured as in Fig 2. Q(NC) and q(similarity) were ploted using the closed PDB ID 1LAF (black line) or the open 2LAO (red line), as reference. Simultaneous changes in the metrics that coincide with crossovers in q(similarity) are indicated by black bars at the bottom of the figure. LAO with *cis* Pro16 (A) displayed two concurrent changes in the metrics whereas with *trans* (B), seven concurrent peaks were detected.

**Fig 7:**
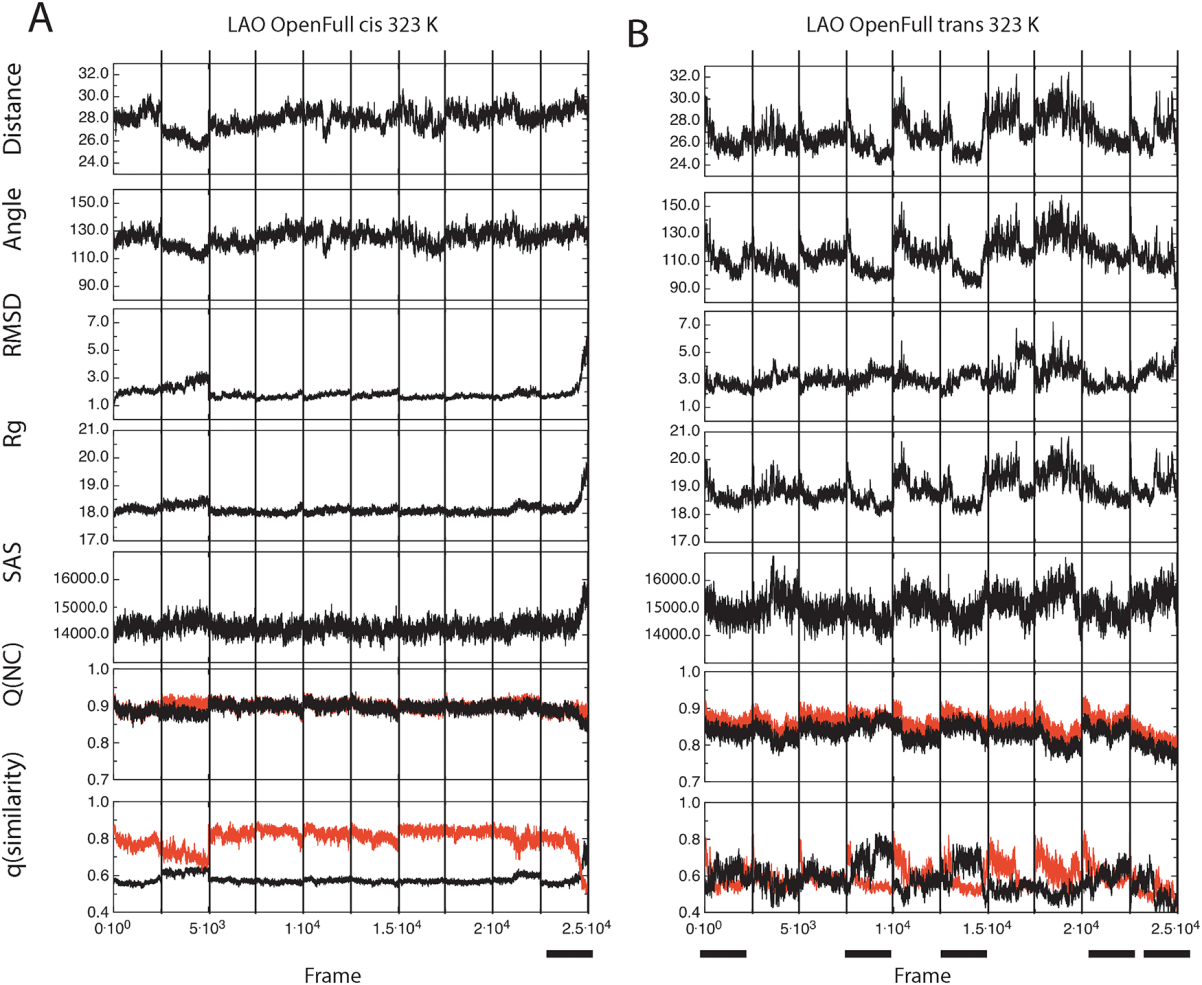
Changes in seven metrics during simulations of the open/with ligand LAO with *cis* or *trans* Pro16 at 323 K. Ten different trajectories were concatenated and metrics were calculated as in Fig 6. Independent trajectories are divided by vertical lines. LAO with *cis* Pro16 (A) displayed one crossover in q(similarity) values, whereas five were detected with *trans* Pro16, concurrent with dips in distance and angle (indicated with black bars at the bottom of the figure).

Once conditions for the simulation of PBP opening and closure were identified, we analyzed concatenated trajectories to identify changes that occurred throughout the simulated time. We simultaneously analyzed distances, angles between domains' centers of mass, RMSD, gyration radius (Rg), solvent accessible surface (SAS), Q(NC), and q(similarity). The aim was to compare these metrics' ability to distinguish PBP opening and closure (dihedrals were not used since in Figs 2 to 5 they showed little sensitivity to detect PBP opening or closure). We focused on states where these events were expected: closed/empty at 300 K (Fig 6 for LAO and S5 Fig for HisJ) and open/with ligand at 323 K (Fig 7 for LAO and S6 Fig for HisJ). Throughout these figures, more changes are evident in all panels with *trans* Pro 16 than with *cis*, compatible with opening or closure being more frequent with the proline in *trans*. By observing all the metrics simultaneously, we propose that peaks in the top five panels correspond to opening events. In turn, closure events are descibed by decreases (valleys) in distance, angle, Rg and SAS, that correlate with peaks in RMSD.

Q(NC) and q(similarity) are shown in the last two panels of Figs 6 and 7, and S5 and S6 Figs. Q(NC) computes the number of contacts between atoms within 8 Å in the simulated vs the native reference structure; while q(similarity) is calculated over all pairs of atoms without a proximity cutoff. Thus, Q(NC) can capture denaturation while q(similarity) can capture domain motion. Q(NC) values oscillated between 0.8 and 0.9 (Figs 6 and 7, and S5 and S6 Figs), suggesting that the native structure was retained during simulations, as shown in S3 and S4 Figs. Previous results with the glutamine binding protein, GBP, suggested that Q could be used to monitor the closed to open transition of PBPs [20]; however, we do not have evidence that this is the case for LAO or HisJ. In contrast, q(similarity) behaved in a way that can aid the identification of the opening and closure of PBPs: crossover between black (closed state reference) and red lines (open state reference) of q(similarity) values, distinguishes events in which trajectories departed from the initial state and approached the other reference. This is noticeable in both LAO figures (Figs 6 and 7). Fig 6 describes open/empty LAO at 300 K. The top five panels displayed 7 concurrent peaks with *trans* Pro16 and two peaks with the proline in *cis*. All the peaks coincided with crossovers in q(similarity) values. With *trans* Pro16: when the reference was the closed state, q(similarity) values started similar to the reference (≈0.85) but became less so (< 0.6) (Fig 6B, bottom panel, black line). When the reference was the open state, the behavior was the opposite: q(similarity) values started off as dissimilar to the open state (< 0.6) but became similar to it (values up to ≈ 0.8) (Fig 6B, bottom panel, red line). In contrast, with *cis* Pro16 (Fig 6A), the simulations departed from the closed state (valleys in black line, bottom panel, from 0.85 to 0.6), but they stayed dissimilar to the open form (red line, from 0.58 to 0.7). So, not only were changes less frequent with *cis* Pro16, but also they presented smaller magnitudes, suggesting that the conformational space accessible to the protein is limited when Pro16 is in *cis*.

LAO trajectories at 323K also displayed more changes in all the metrics with *trans* than with *cis* Pro16 (Fig 7B vs 7A). With *trans* Pro16, distance and angles between domains displayed valleys in which the initially open structure approached the values of the crystallographic closed reference (Fig 7B, top two panels). These valleys did not clearly correspond to peaks in RMSD, Rg or SAS, which became noisier with *trans* Pro16 at this temperature (Fig 7B, middle panels). However, they corresponded with frames where q(similarity)_closed_ crossed-over q(similarity)_open_ values (Fig 7, bottom panels), suggesting 5 trajectories that closed with *trans* Pro16, but just one with *cis* (marked by black horizontal bars).

Results for HisJ were similar to LAO: more changes in all the metrics were seen with *trans* than with *cis* Pro16, particularly at 300 K. Both at 300 and 323 K, the red line of q(similarity)_open_ crossed-over the black line of q(similarity)_closed_ more times with *trans* than with *cis* Pro16, marking more putative opening and closure events with *trans* (black bars at the bottom of S5 and S6 Figs). For HisJ at 323 K, the changes in q(similarity) values were small, resulting in the black and red lines almost overlapping through part of the trajectories (S6 Fig) and making crossovers hard to identify. This suggests that HisJ and LAO may behave differently in the open/close transition and further analyses are needed to explore this.

## Discussion

PBPs have been widely studied as biophysical models for proteins exhibiting large conformational changes, but a complete atomistic understanding of the determinants that open and close the PBP flytrap is missing. In this paper we simulated two steps of the cycle of amino acid capture and release for the ortholog PBPs, LAO and HisJ: opening in the absence of ligand and closure in its presence. While opening was detectable at 300 and 323 K, closure was only detectable at 323 K. Denaturation was discarded as a component of the conformational changes observed at either temperature. Notably, PBP opening and closure were more frequent and evident when the conserved proline 16 was isomerized from *cis* to *trans* (S7 Fig). This residue is near the binding site but does not contact the ligand. Our results suggest that the isomerization of this conserved proline modulates the propensity of the trap to open and close in the amino acid binding PBPs, LAO and HisJ. Data presented here suggest that when Pro16 is in *trans*, the proteins explore more conformational space and are more prone to open or close upon a ligand.

The conservation of the studied proline, suggests that trap control through Pro isomerization could be widespread among PBPs. Spontaneous *cis*-*trans* proline isomerization is slow, in the order of microseconds to seconds and could thus act as a point of kinetic control with ample oportunities for regulation. A few aromatic residues in the vicinity of Pro16 are also conserved, including the binding site residue, Tyr 14. From the HisJ 3D structures it is obvious that the isomerization state of Pro16 has a large impact on the orientation of Tyr14 (Fig 1C and 1D). A change in the charge distribution of Tyr14 upon ligand binding could modulate Pro16's isomerization rate. Ligand binding could limit the degrees of freedom of Tyr14 and the nearby backbone, favoring the isomerization of Pro16 to *cis*, to retard PBP opening. In peptides, neighboring tyrosines decrease proline's isomerization rate, favoring *cis* [21]. Our results suggest that this isomerization could happen once the PBP has closed upon the ligand, as *trans* Pro16 favors PBP closure. Thus, we propose a working model where *trans* Pro 16 favors PBP closure upon ligand binding. Ligand bindng, in turn would favor Pro16 isomerization to *cis*, aided by Tyr14 and/or the aromatic residues in the vicinity, to keep the trap closed until ligand release is triggered into the ABC transporter. Isomerization of Pro 16 back to *trans* would accelerate ligand release and cycle the protein back to the ligand binding state.

Proline isomerization has been associated with protein folding [22] and more recently, with signal transduction and ion channel opening [23], but this is the first time such isomerization has been related to a large conformational change like the opening/closure mechanism of the PBP flytrap. The bacterial periplasm, where PBPs reside, is rich in prolyl-isomerases that play roles in coordinating survival in pathogenic niches and stressful conditions [24,25]. In this environment, the restriction to open the PBP flytrap by a *cis* proline, could add a useful layer of control to the process of substrate release. Prolyl isomerases could ensure that the nutrients are only released until productive docking into the ABC transporter. Furthermore, the closed with ligand form of the PBPs would lower the effective concentration of the nutrient in the periplasm, thus stimulating diffusion. Once the PBPs are bound to the ABC, the mechanisms that may couple PBP opening and ligand release into the ABC transporter are unknown. Our data open the unexplored possibility that the ABC transporter may have prolyl-isomerase activity or that there is a PBP-prolyl-isomerase complex as of yet unidentified.

Q(NC) values were not able to distinguish the open from the closed states as previously shown for Gō model simulations [20]. It is noteworthy that for the all-atom simulations described here, q(similatiry) was capable of detecting departures from the closed state that became similar to the open and viceversa. This suggests that q(similarity) could improve the sensibility of other metrics to detect PBP opening/closure and conformational changes in proteins.

Data presented here uncover a piece of the PBP flytrap mechanism that needs further exploring and that may be key for the atomistic understanding of binding and transport of nutrients in this bacterial system. Finally, it is important to point out that the many molecular dynamics simulations aimed at understanding PBP's conformational changes have been performed without taking into account Pro16 isomerization state and should be reevaluated in view of these results.

## Methods

PBP homologous sequences were obtained using CSI-BLAST [26] over the Uniref-90 [27] with a maximum identity of 95% and a minimum of 30% followed by alignment with MAFFT-L-INS-I [28]. PDBs were obtained from the RCSB website and their stereochemistry was checked using Molprobity (see S7 Fig for a summary) [29]. The following PBP structures were used for simulation: PDB IDs 2LAO and 1LAF for LAO [14] and PDB IDs 2M8C [15] and 1HSL [11] for HisJ. 2LAO and 2M8C are open states; 1LAF and 1HSL correspond to closed/with ligand and contain arginine (LAO) or histidine (HisJ) at the binding site. 2M8C was solved from NMR while all other structures are XRD. While the structures used in this study were produced before electron density data deposition was compulsory; the presence of a cis-proline in position 16 is by no means unique to PBP structures: there are over 50 related structures listed in the RCSB data base that contain the same cis-proline supported by electron density data. This makes it unlikely that the cis-proline in the closed LAO and HisJ structures used in this work are derived from an error in structure refinement.

All water molecules were computationally removed before simulations. To simulate empty PBPs ligands were removed. 1LAF (LAO) is missing sidechains for several residues: 5 lysines, 1 glutamate and 1 glutamine; all were reconstructed in AMBER’s tleap independently for each molecular dynamics (MD) run. The isomerization state of Proline 16 (Pro 16) was checked using VMD’s “Fix cis peptide bonds” plugin [30]. Changes in the isomerization state were performed using Coot [31], verified after processing on AMBER’s tleap [32,33], and before and after MD production runs. No spontaneous proline isomerizations were observed during simulations.

Molecular dynamics were run in AMBER14. Structures were prepared by indicating the disulfide bond present in its native form between cysteine residues 38 and 45. Since LAO and HisJ both have aminoacids as ligands, there was no need to parameterize them and they were automatically handled by tleap. Simulations were ran using TIP3P water model, with AMBER14SB [34] force field for the protein and ligands. The proteins were solvated in an octahedral water box with a minimal 10 Å distance from the protein surface to the box edge; the proteins were neutralized with Na+. Once solvated and neutralized, the water molecules were first relaxed while the protein was restrained with a force constant of 500 kcal/mol Å^2^. Then, restrains were released and the solvent and solute were both relaxed. In both instances, minimization consisted of 5000 simulated anneling-steps, followed by 5000 conjugated gradient-steps; PME cutoff was set at 10 Å. The temperature of the system was slowly raised from 0 to 300K or 323K while a 10 kcal/mol Å^2^ constant force restrained the complex; from this step on, a Langevin thermostat was employed with a collision frequency of 1 ps. Time step was set to 1 fs and SHAKE constrains for bonds involving hydrogens were turned on. This minimization step improved some sterochemical parameters as shown in S7 Fig. Constant pressure coupling, with relaxation time of 2 ps, was introduced while the restrains were released and the simulation was extended for 200 to 500 ps; isotropic scaling was employed from this step onward. After this step, 50 ns production runs were performed. Each simulation was minimized, equilibrated and production-ran independently.

Simulation analysis was carried out using cpptraj [35] with additional bash scripting and visualized with VMD. All 3D structure figures were prepared using UCSF Chimera [36]. For distance and angle analyses, domain 1 was defined as residues 1–86 and 197–238; domain 2 as residues 90–192 and the hinge as residues 87–89 and 193–196 (two linkers), based on HisJ nomenclature [10]. Box and whisker plots were crafted in Graphpad Prism 6.00 for OS X, GraphPad Software, La Jolla California USA (www.graphpad.com). Evaluation of the native contacts and structural clustering (Q(NC) and q(similarity) values) was done using carma [37] via its GUI Grcarma [18] using Cα with the open or closed state as reference, using the following equations:

$$Q(NC)=\frac{1}{\left(NC-1)(NC-2\right)}\sum_{}^{NC}exp[-\frac{{\left(r_{ij}^{nat}{-r}_{ij}^{comp}\right)}^{2}}{{2\sigma }_{ij}^{2}}]$$

Where *NC* stands for the number of native contacts while r_ij_^nat^ and r_ij_^comp^ are the distances between residues i and j in the native and comparison structures, respectively. σ is the width of the Gaussian:

$$\sigma ={\left|i-j\right|}^{0.15}$$

For q(similarity):

$$q(similarity)=\frac{1}{\left(n-1)(n-2\right)}\sum_{i<j-1}^{n}exp[-\frac{{\left(r_{ij}^{ref}-r_{ij}^{comp}\right)}^{2}}{\sigma_{ij}^{2}}]$$

Where *n* stands for the native contacts for the reference structure [16–18]; here r_ij_^ref^ and r_ij_^comp^ are the distances between residues i and j in the reference and comparison structures, respectively. These equations should not be confused with the one reported in [38]. For Q(NC) calculations a cutoff of 8 Å and a residue distance of 2 were employed.

Binning and other preprocessing were done using cpptraj to 100 bins, limited to values between 0.4 and 1 and graphed with QuantumSoft’s Pro Fit (www.quansoft.com). All structural calculations were performed after structural alignment using all heavy atoms. Gyration radius and surface accessible areas were calculated for all heavy atoms whereas RMSD, Q(NC) and q(similatiry) only considered Cα.

## Supporting information

S1 Table Number of simulations per condition: Temperature, Pro16 isomerization state, open or closed conformation and presence or absence of ligand. Each simulation was 50 ns long.
(DOCX)

S1 Fig Centers of masss employed to measure distance, angle and dihedral, shown in open and closed LAO 3D models. Centers of mass are indicated by color spheres joined by black lines. A comparison between the distances, angle and dihedral for the open 2LAO structure in beige (A, B and C) are shown side by side to those for the closed 1LAF in green (D, E and F). The residues used to define the centers of mass are described in the Methods section.
(TIF)

S2 Fig RMSD values for representative LAO simulations that displayed opening or closure. LAO simulations that started in the closed/empty (A and B) or open/with ligand (C and D) states, with *cis* Pro16 (cyan, red) or *trans* Pro16 (green, yellow) were followed by RMSD (A and C) and the final structures are shown (B and D). RMSD values are different throughout the simulation and larger with *trans*. Only with *trans* Pro16, LAO opens (green in B) or closes (yellow in D). A black bar has been drawn alongside helix 8 to emphasize the diferent conformations: An almost vertical bar identifies an open structure while one with a negative slope shows a closed structure.
(TIF)

S3 Fig Evaluation of LAO's native state during simulations at 300 and 323 K through Q(NC) analyses. Plots depict Q(NC)open vs Q(NC)closed for simulations with *cis* or *trans* Pro16, starting from the open/empty (A, E, I, M), closed/empty (B, F, J, N), closed/with ligand (C, G, K, O) or open/with ligand (D, H, L, P) states. Each trajectory was processed independently in grcarma and then binned together before plotting. Colors depict the frequencies in a logarithmic scale, base 10. Native state references were the open PDBID 2LAO for Q(NC)open and the closed 1LAF for Q(NC)closed. A value of 1 is identical to native state, whereas decreasing values describe decreasing similarity. Q(NC) values remained similar to both the open and closed native states, well above 0.7, irrespective of temperature or Pro16 isomer, suggesting no denaturation, even in cases where conformational changes were detected by other metrics.
(TIF)

S4 Fig Evaluation of HisJ’s native state during simulations at 300 and 323 K through Q(NC) analyses. Plots depict Q(NC)open vs Q(NC)closed for simulations with *cis* or *trans* Pro16 starting from the open/empty (A, E, I, M), closed/empty (B, F, J, N), closed/with ligand (C, G, K, O) or open/with ligand (D, H, L, P) states. Trajectory processing was as in S3 Fig. Native reference states were PDBIDs 2M8C for Q(NC)open and 1HSL for Q(NC)closed. Q(NC) values remained similar to the open and the closed native states, suggesting that no denaturation occurred, irrespective of temperature or Pro16 isomer.
(TIF)

S5 Fig Changes in seven metrics during simulations of the closed/empty state for HisJ with *cis* or *trans* Pro16 at 300 K. Ten different trajectories were concatenated and changes in distance, angle, RMSD, Rg, SAS, Q(NC) and q(similarity) were calculated and plotted. Distances and angles were measured as in Fig 3. Q(NC) and q(similarity) were ploted using the closed 1HSL (black line) or the open 2M8C state (red line), as reference. Crossovers in q(similarity) coinciding with changes in other metrics are indicated by black bars at the bottom the figure. No crossovers in q(similarity) ocurred with *cis* Pro16 (A), while with *trans* Pro16 (B) three crossovers with simultanoeus changes in other metrics were detected.
(TIF)

S6 Fig Changes in seven metrics during simulations of the closed/empty state for HisJ with *cis* or *trans* Pro16 at 323 K. Ten different trajectories were concatenated and changes in distance, angle, RMSD, Rg, SAS, Q(NC) and q(similarity) were calculated and plotted as in S7 Fig, with the same references for Q(NC) and q(similarity). Three crossovers in q(similarity) ocurred with *cis* Pro16 (A), while with *trans* Pro16 (B) four crossovers were detected.
(TIF)

S7 Fig Diagram of the effect of Pro 16 isomerization state on PBP conformational change. Opening of empty (A) and closure of open/with ligand PBPs (B) is favored by Pro16 in *trans*.
(TIF)

S8 Fig Comparison of stereochemical parameters calculated in Molprobity for the structures used, before and after the minimization step. (A) Clashscore, (B) Ramachandran outliers and (C) beta carbon deviations above 0.25 angstroms. XRD structures are presented in black and named according to the PDB/ID; minimized structures at 300 K are shown in red and those minimized at 323 K are in blue. The scale of the y-axis in all cases is set to one residue. This analysis highlights that energy minimization relieves the atom clashes within structures, reduces Ramachandran outlayers as well as beta-carbon deviations. Thus, the quality of the structures is suitable for molecular dynamics.
(TIF)
