# Supplementary material for: Role of cis-trans proline isomerization in the function of pathogenic enterobacterial Periplasmic Binding Proteins
Source: PLoS One. 2017 Nov 30;12(11):e0188935. doi: 10.1371/journal.pone.0188935 (PMC5708682; doi:10.1371/journal.pone.0188935)
Supplement: S1 Table — Each simulation was 50 ns long. (DOCX) [file pone.0188935.s001.docx]

S 1 Table. Number of simulations per condition: temperature, Pro16 isomerization state, open or closed conformation and presence or absence of ligand. Each simulation was 50 ns long.

|  |  | LAO (ArgT) | | HisJ | |
| --- | --- | --- | --- | --- | --- |
| 300 K |  | Open (2LAO) | Closed (1LAF) | Open (2M8C) | Closed (1HSL) |
| With ligand | *cis Pro16* | 10 | 10 | 10 | 10 |
|  | *trans Pro16* | 10 | 10 | 10 | 10 |
| Without ligand | *cis Pro16* | 10 | 10 | 10 | 10 |
|  | *trans Pro16* | 10 | 10 | 10 | 10 |
| 325 K |  |  |  |  |  |
| With ligand | *cis Pro16* | 10 | 10 | 10 | 10 |
|  | *trans Pro16* | 10 | 10 | 10 | 10 |
| Without ligand | *cis Pro16* | 10 | 10 | 10 | 10 |
|  | *trans Pro16* | 10 | 10 | 10 | 10 |
