# Supplementary figures and images for: Role of cis-trans proline isomerization in the function of pathogenic enterobacterial Periplasmic Binding Proteins
Source: PLoS One. 2017 Nov 30;12(11):e0188935. doi: 10.1371/journal.pone.0188935 (PMC5708682; doi:10.1371/journal.pone.0188935)

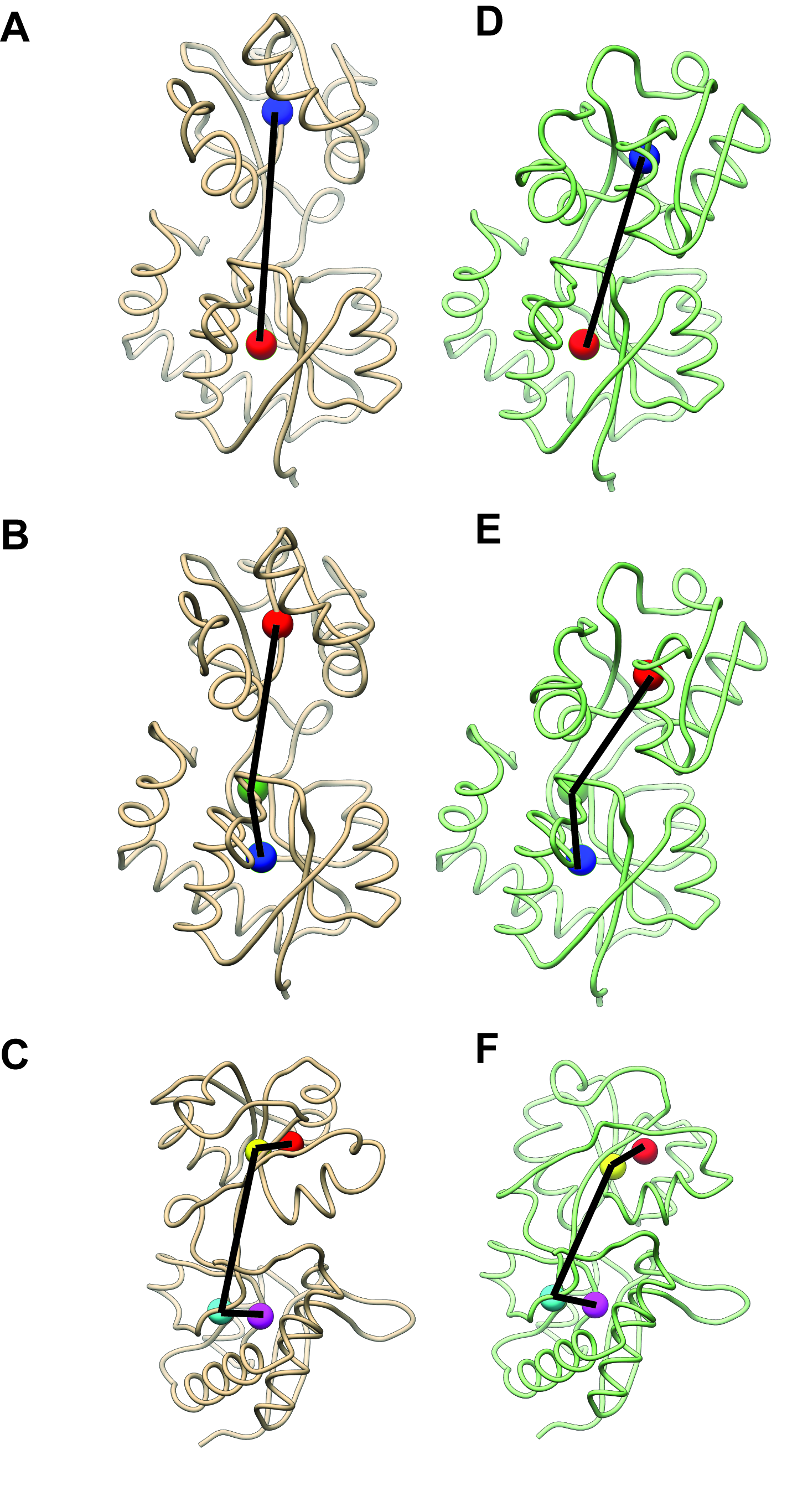

Supplement: S1 Fig — Centers of mass are indicated by color spheres joined by black lines. A comparison between the distances, angle and dihedral for the open 2LAO structure in beige (A, B and C) are shown side by side to those for the closed 1LAF in green (D, E and F). The residues used to define the centers of mass are described in the Methods section. (TIF) [file pone.0188935.s002.tif]

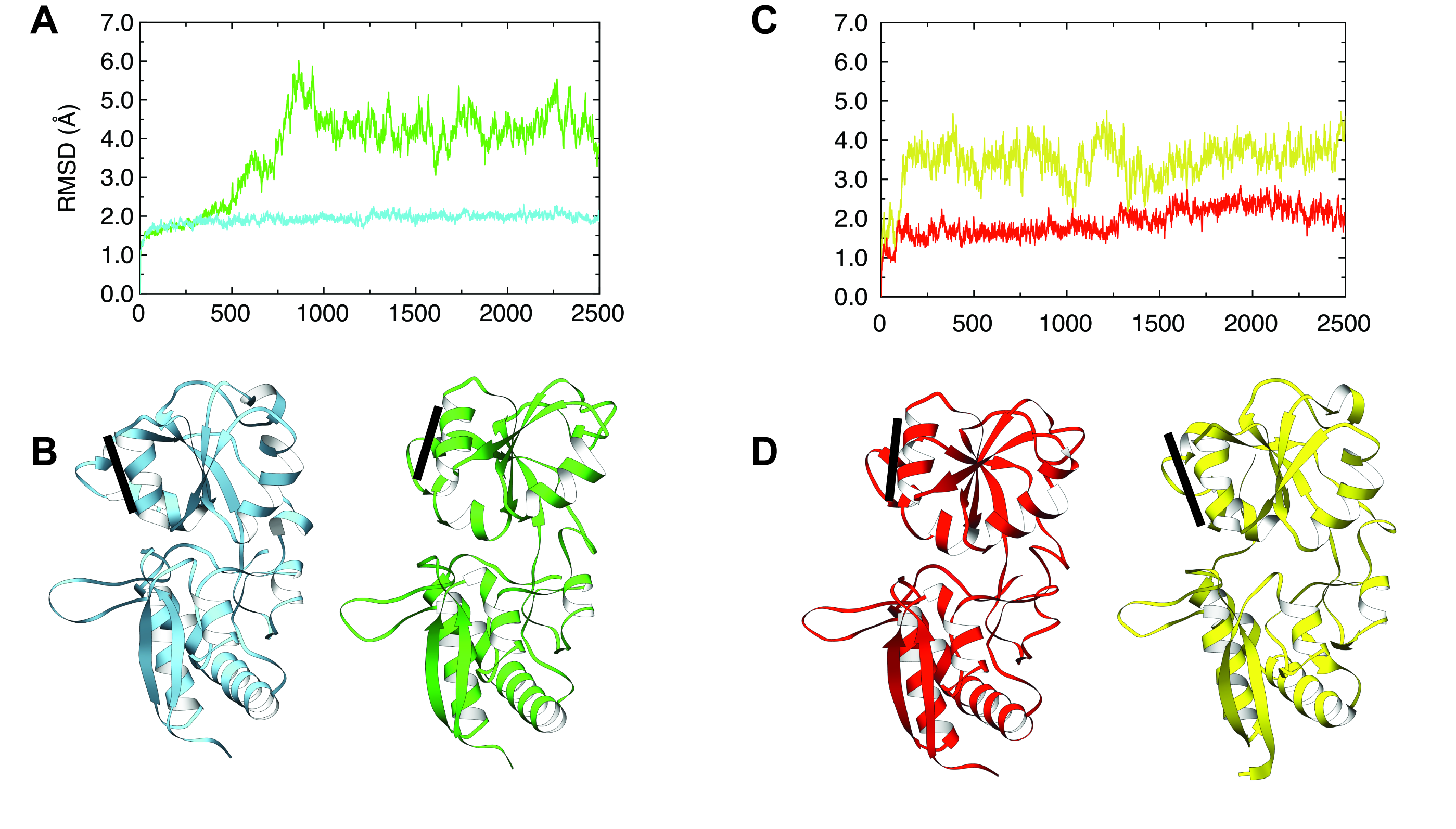

Supplement: S2 Fig — LAO simulations that started in the closed/empty (A and B) or open/with ligand (C and D) states, with cis Pro16 (cyan, red) or trans Pro16 (green, yellow) were followed by RMSD (A and C) and the final structures are shown (B and D). RMSD values are different throughout the simulation and larger with trans. Only with trans Pro16, LAO opens (green in B) or closes (yellow in D). A black bar has been drawn alongside helix 8 to emphasize the diferent conformations: An almost vertical bar identifies an open structure while one with a negative slope shows a closed structure. (TIF) [file pone.0188935.s003.tif]

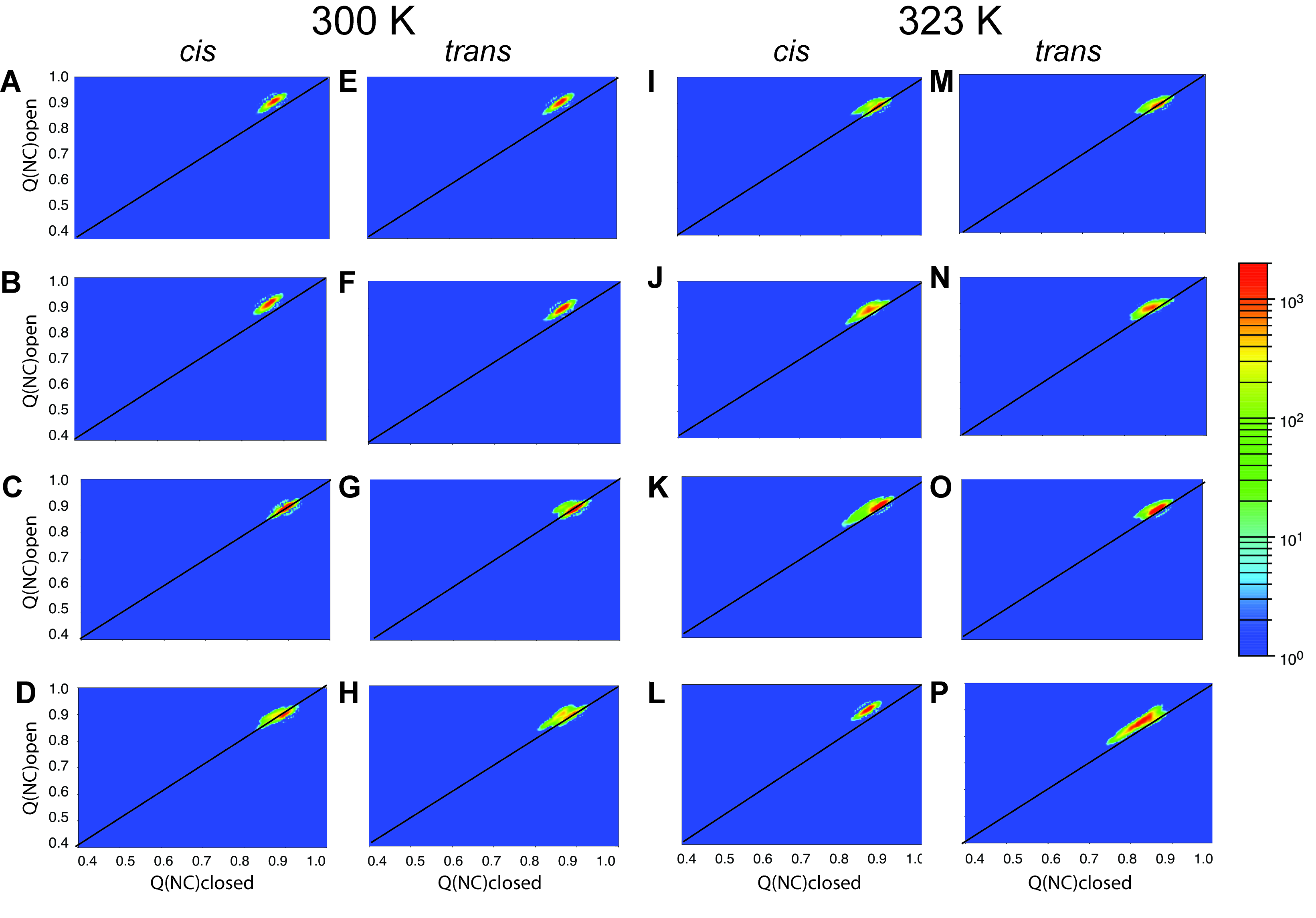

Supplement: S3 Fig — Plots depict Q(NC)open vs Q(NC)closed for simulations with cis or trans Pro16, starting from the open/empty (A, E, I, M), closed/empty (B, F, J, N), closed/with ligand (C, G, K, O) or open/with ligand (D, H, L, P) states. Each trajectory was processed independently in grcarma and then binned together before plotting. Colors depict the frequencies in a logarithmic scale, base 10. Native state references were the open PDBID 2LAO for Q(NC)open and the closed 1LAF for Q(NC)closed. A value of 1 is identical to native state, whereas decreasing values describe decreasing similarity. Q(NC) values remained similar to both the open and closed native states, well above 0.7, irrespective of temperature or Pro16 isomer, suggesting no denaturation, even in cases where conformational changes were detected by other metrics. (TIF) [file pone.0188935.s004.tif]

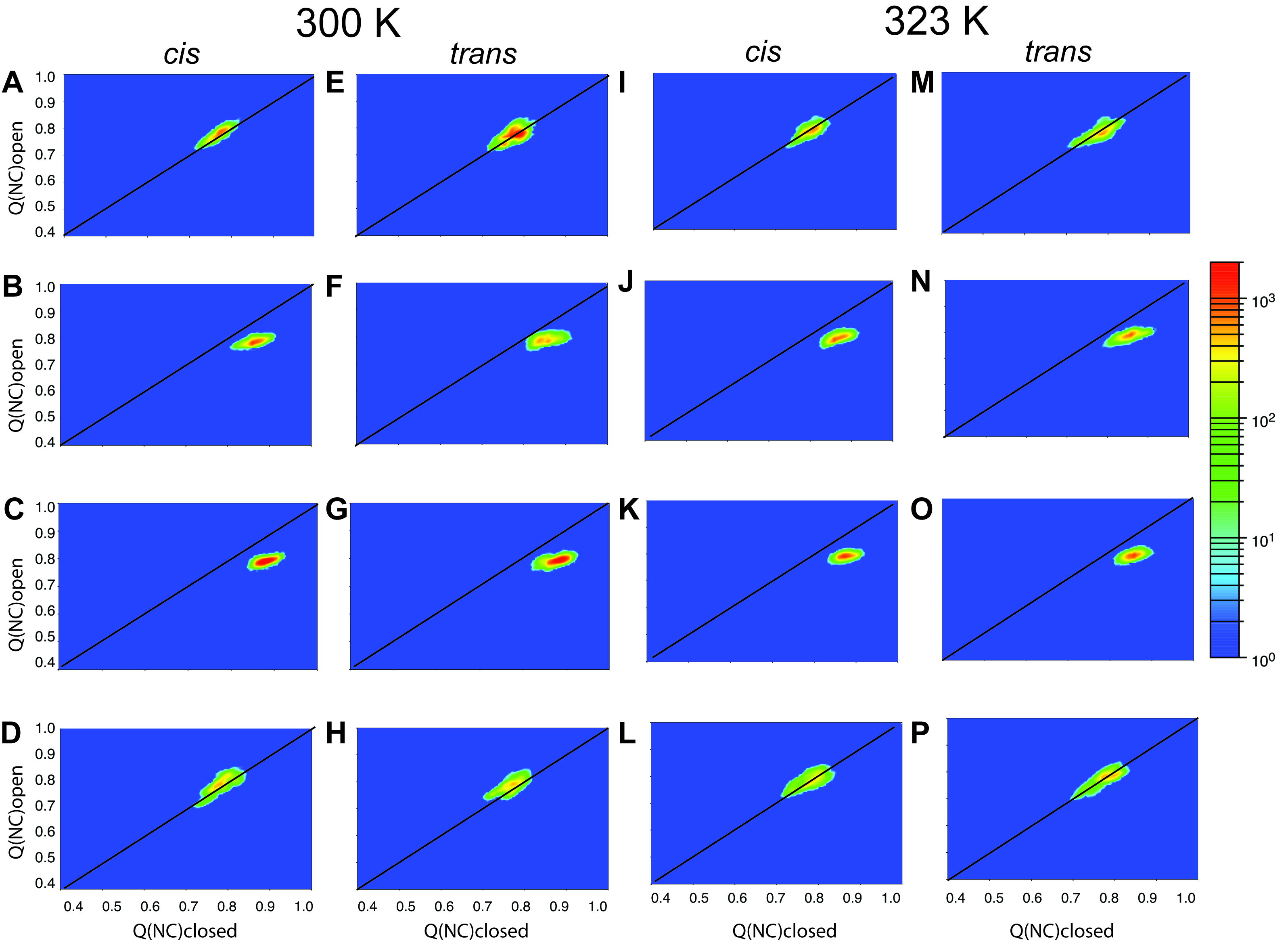

Supplement: S4 Fig — Plots depict Q(NC)open vs Q(NC)closed for simulations with cis or trans Pro16 starting from the open/empty (A, E, I, M), closed/empty (B, F, J, N), closed/with ligand (C, G, K, O) or open/with ligand (D, H, L, P) states. Trajectory processing was as in S3 Fig. Native reference states were PDBIDs 2M8C for Q(NC)open and 1HSL for Q(NC)closed. Q(NC) values remained similar to the open and the closed native states, suggesting that no denaturation occurred, irrespective of temperature or Pro16 isomer. (TIF) [file pone.0188935.s005.tif]

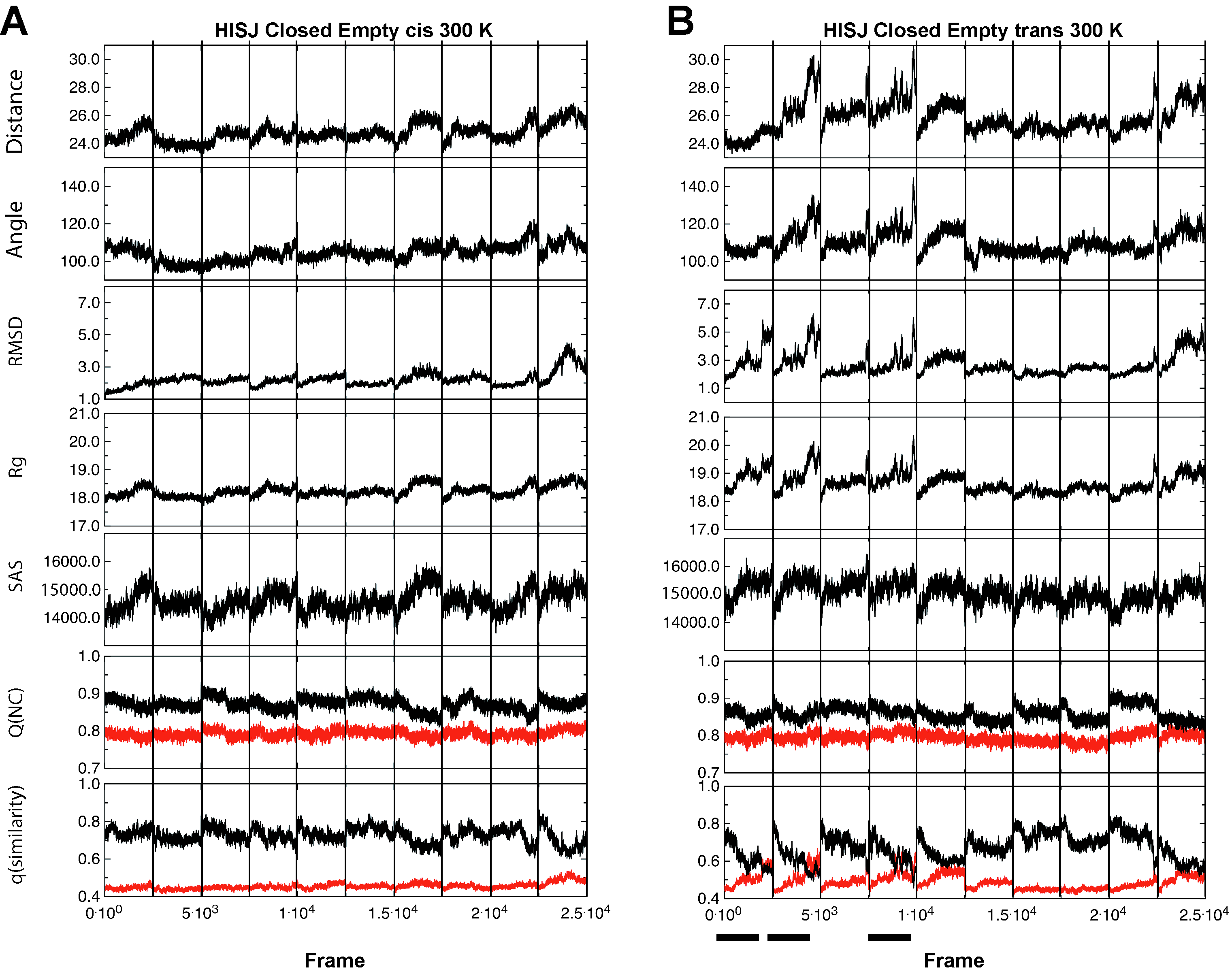

Supplement: S5 Fig — Ten different trajectories were concatenated and changes in distance, angle, RMSD, Rg, SAS, Q(NC) and q(similarity) were calculated and plotted. Distances and angles were measured as in Fig 3. Q(NC) and q(similarity) were ploted using the closed 1HSL (black line) or the open 2M8C state (red line), as reference. Crossovers in q(similarity) coinciding with changes in other metrics are indicated by black bars at the bottom the figure. No crossovers in q(similarity) ocurred with cis Pro16 (A), while with trans Pro16 (B) three crossovers with simultanoeus changes in other metrics were detected. (TIF) [file pone.0188935.s006.tif]

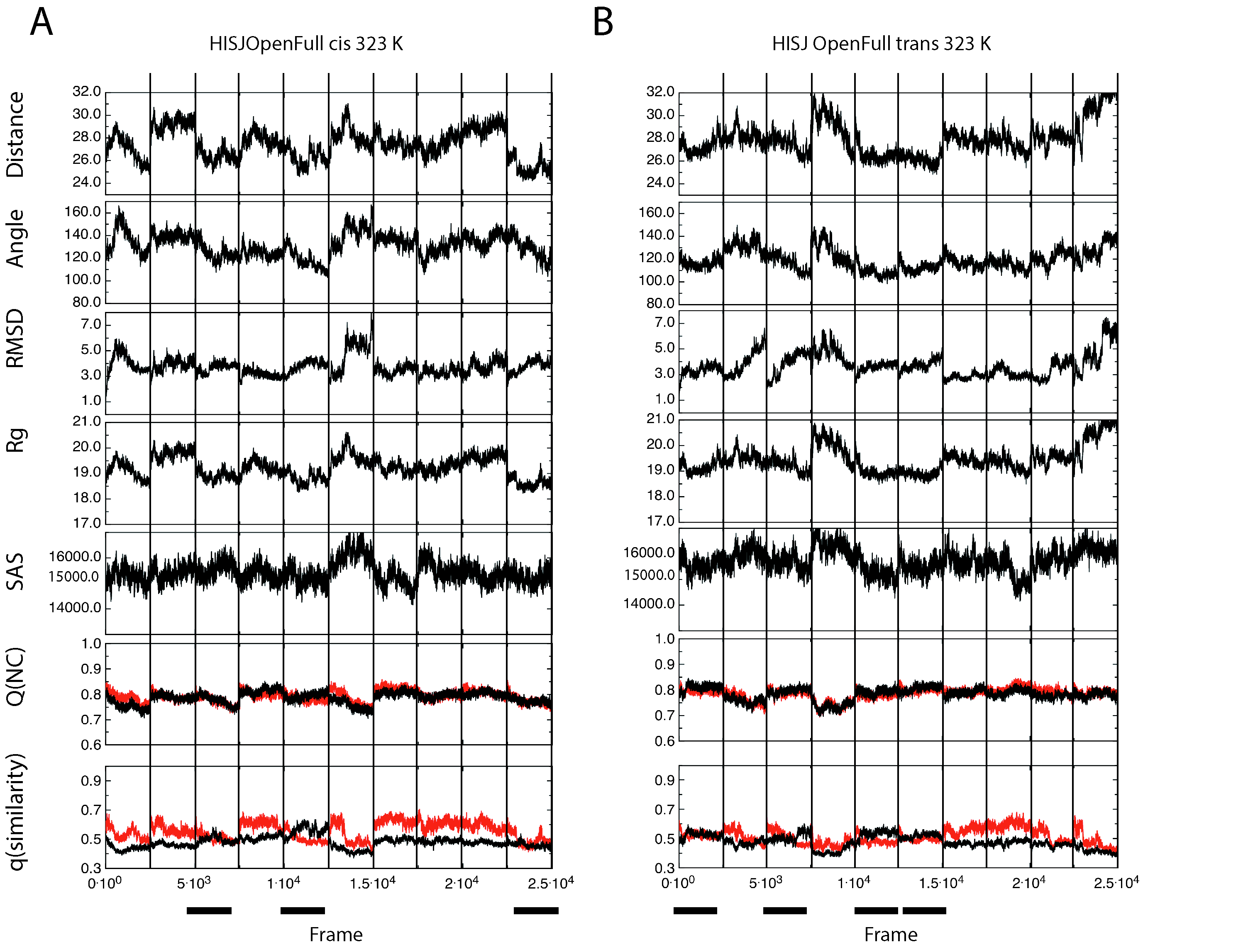

Supplement: S6 Fig — Ten different trajectories were concatenated and changes in distance, angle, RMSD, Rg, SAS, Q(NC) and q(similarity) were calculated and plotted as in S7 Fig, with the same references for Q(NC) and q(similarity). Three crossovers in q(similarity) ocurred with cis Pro16 (A), while with trans Pro16 (B) four crossovers were detected. (TIF) [file pone.0188935.s007.tif]

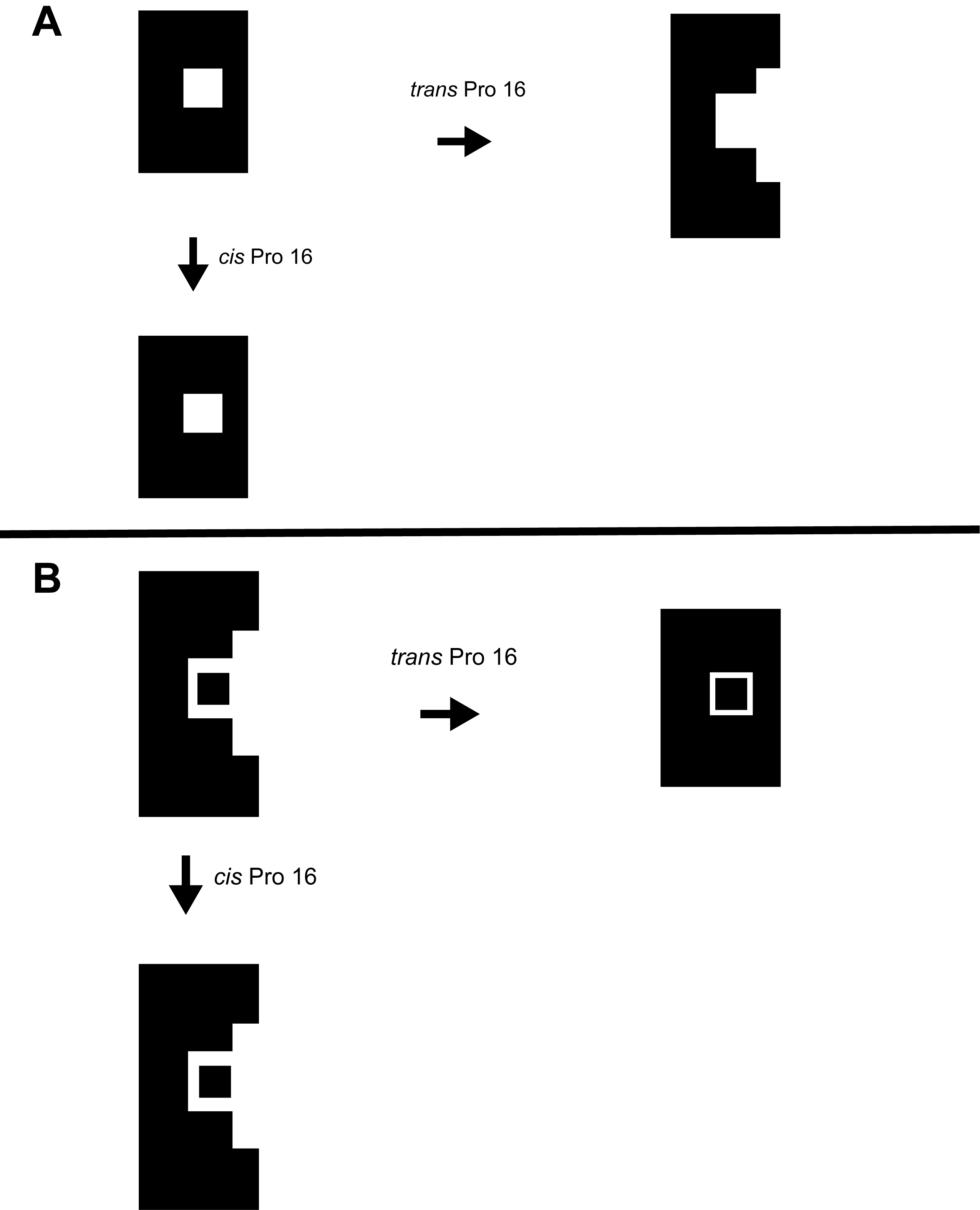

Supplement: S7 Fig — Opening of empty (A) and closure of open/with ligand PBPs (B) is favored by Pro16 in trans. (TIF) [file pone.0188935.s008.tif]

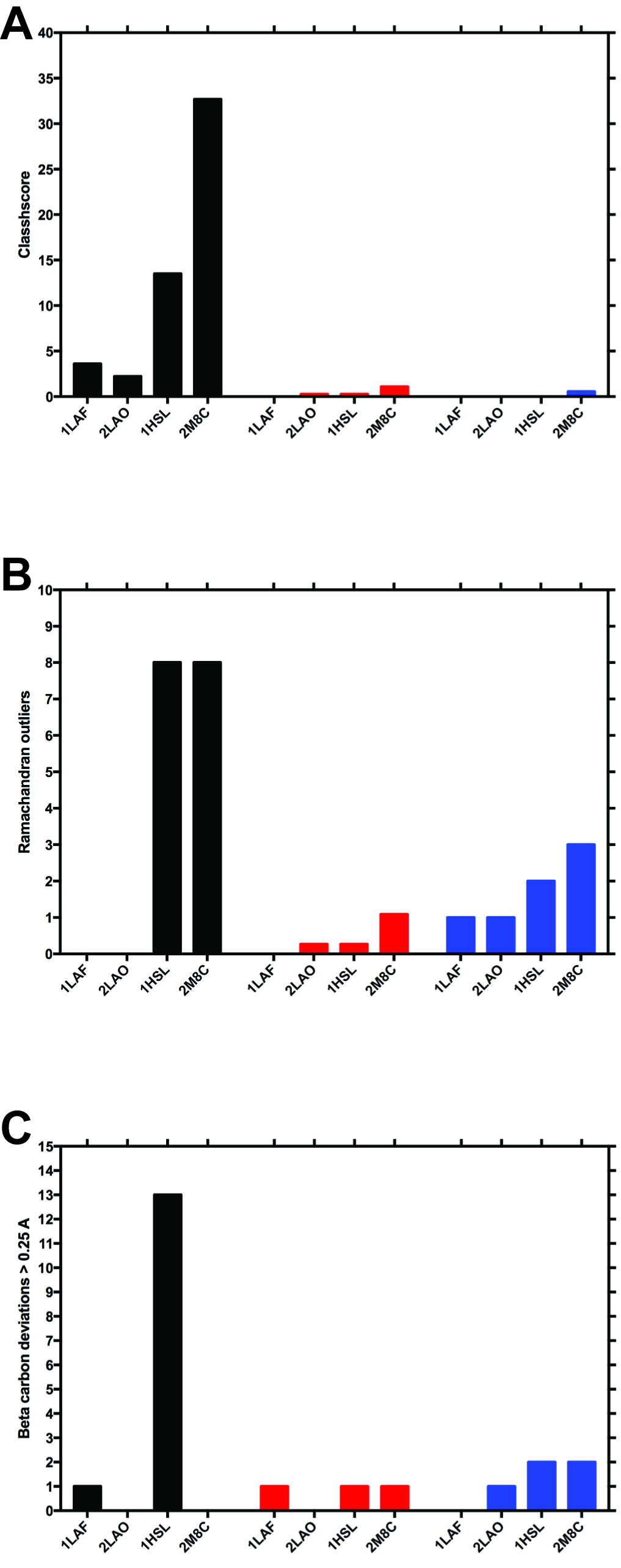

Supplement: S8 Fig — (A) Clashscore, (B) Ramachandran outliers and (C) beta carbon deviations above 0.25 angstroms. XRD structures are presented in black and named according to the PDB/ID; minimized structures at 300 K are shown in red and those minimized at 323 K are in blue. The scale of the y-axis in all cases is set to one residue. This analysis highlights that energy minimization relieves the atom clashes within structures, reduces Ramachandran outlayers as well as beta-carbon deviations. Thus, the quality of the structures is suitable for molecular dynamics. (TIF) [file pone.0188935.s009.tif]
